# Survey of *Salmonellae* occurrence in meat‐producing rabbitries in Switzerland

**DOI:** 10.1002/vro2.24

**Published:** 2022-01-22

**Authors:** Julia Schädler, Julia Schwarz, Judith Peter‐Egli, Gertraud Schüpbach‐Regula, Danja Wiederkehr, Sarah Albini

**Affiliations:** ^1^ National Reference Centre for Poultry and Rabbit Diseases (NRGK) Institute for Food Safety and Hygiene Vetsuisse Faculty University of Zurich Zurich Switzerland; ^2^ School of Agricultural Forest and Food Sciences Berne University of Applied Sciences Zollikofen Switzerland; ^3^ Swiss Association of Swine Medicine (SVSM) Herbligen Switzerland; ^4^ Veterinary Public Health Institute Vetsuisse Faculty University of Berne Liebefeld Switzerland

**Keywords:** animal welfare‐friendly housing, meat rabbits, Salmonella Typhimurium, whole genome sequencing

## Abstract

**Background:**

An outbreak of salmonellosis due to *Salmonella* Typhimurium was detected coincidentally in a Swiss meat rabbitry, given that surveillance of *Salmonella* in rabbits is not mandatory in Switzerland.

**Methods:**

To assess the extent of potentially subclinical *Salmonella* carriage in meat rabbits, faecal pool samples of 50 farms (90% of Swiss commercial rabbitries) with ground covering litter and group housing were bacteriologically tested. Additionally, 236 rabbits showing clinical signs compatible with intestinal diseases, such as salmonellosis, were examined postmortem and analysed bacteriologically. *Salmonella* isolates were serotyped and analysed by whole genome sequencing (WGS).

**Results:**

*Salmonella* Typhimurium was detected in three commercial farms (6.0% of all tested farms). The affected farms were directly linked to the animal trade and *Salmonella* isolates were shown to be identical by WGS.

**Conclusion:**

There is no increased hazard for *Salmonella* carriage in the animal welfare‐friendly Swiss husbandry systems in general, despite risk factors such as ground covering litter.

## INTRODUCTION

In 2018, an outbreak of salmonellosis was detected on a Swiss meat rabbitry because of increased mortality rates in fattening and breeding rabbits. Rabbits of all types and ages, with or without clinical signs, were tested positive for *Salmonella enterica* subsp. *enterica* serovar Typhimurium (*Salmonella* Typhimurium). While *Salmonella* Typhimurium occasionally causes severe outbreaks of salmonellosis with fever, enteritis, abortion and increased mortality,^1^, ^2^, ^3^, ^4^, ^5^ rabbits may also be subclinical carriers of several *Salmonella* serovars.^2^, ^6^, ^7^, ^8^, ^9^ *Salmonella* carriage in clinically healthy livestock can result in contamination of carcasses at slaughter and thereby poses a hazard for meat‐borne infections in humans.^10^, ^11^, ^12^ In the European Union, salmonellosis ranks second in zoonoses in humans with *Salmonella* Enteritidis and *Salmonella* Typhimurium being the two serovars with the highest prevalence.^13^ *Salmonella* control programmes implemented in Switzerland mainly focus on poultry, cattle and pigs. They do not consider less abundant food‐producing species, such as meat rabbits. While two studies, focussing on suckling mortality^14^ and on faecal samples of rabbits at slaughter,^11^ did not detect *Salmonella* in Swiss rabbits. The current study, with reference to the findings in Farm A, raised the question, whether the extent of *Salmonella* carriage in commercial rabbits might be underestimated.

In commercial rabbit husbandries in Switzerland, housing on wire mesh floors and single housing of fattening rabbits at the age of less than 8 weeks is prohibited.^15^ In this study, the participating farms represent the majority of the commercial production in Switzerland, with husbandry systems exceeding the minimal requirements of the legislation, providing ground covering litter for all rabbits and partly group housing for breeding does.^16^, ^17^ These high‐level housing conditions regarding animal welfare may be challenging concerning hygiene management and the prevention of disease transmission. Hence, the objective of this study was to assess the occurrence of *Salmonella* in Swiss commercial rabbitries under these animal welfare‐friendly husbandry conditions.

## MATERIAL AND METHODS

### History of *Salmonella* positive farm (farm A)

The initial detection of *Salmonella* Typhimurium on farm A was based on nine submissions of rabbits for postmortem examination at the National Reference Centre for Poultry and Rabbit Diseases (NRGK) in October 2018. During subsequent sanitation efforts on the farm, faecal and environmental samples were examined. Resulting *Salmonella* isolates were stored.

### Study farms and faecal sample collection

A total of 50 Swiss commercial rabbitries were tested for *Salmonella* (Table 1). At least 60 g of fresh faeces were collected randomly in at least five different pens (both breeding and fattening) on each farm. During the first sampling period (April–December 2019), rabbits were assessed on‐site for clinical signs consistent with salmonellosis by the same veterinarian; husbandry and management data were collected. Rabbitries initially testing negative were re‐tested in a second sampling period (February–August 2020). On positive farms, animals other than rabbits were also tested to exclude cross‐contamination by methods authorized by the Swiss Federal Veterinary Office.^18^, ^19^

**TABLE 1:** Production data of 50 Swiss rabbitries (nine breeding farms, 13 fattening farms and 28 combined farms) and results of pathological and microbiological examination of rabbits originating from *Salmonella* Typhimurium‐positive farms A, B and C

		***Salmonella* Typhimurium positive rabbitries**			***Salmonella* Typhimurium negative rabbitries**
**Farms**		**Farm A**	**Farm B**	**Farm C**	**Other farms (*n* = 47)**
*Salmonella* Typhimurium isolates [sample designation]		Faecal and environmental samples, organs [101, 143, 170, 257, 261, 288, 514, 540, 547]	Pooled faecal sample of both breeding and fattening unit [N19‐2115/19‐S3101]	Pooled faecal sample [N19‐2269/19‐S3402]	n.a.
**Husbandry**					
Breeding does	Number	560	250	0	3,586 animals (6‐360 per farm)
	Continuous, group housing	yes	yes	n.a.	34% (12/35)
	Single housing system	no	no	n.a.	66% (23/35)
Fattening rabbits	Number	10,000	1000	1200	36,348 animals (120–4000 per farm)
	All‐in/all‐out, group housing	yes	yes	yes	29% (11/38)
	Continuous, group housing	no	no	no	71% (27/38)
	Mortality^1^	25.29%	14.10%	16.17%	n.a.
**Hygiene**					
Regular disinfection of pens	Breeding units	no	no	n.a.	yes: 37% (13/35)
	Fattening units	yes	yes	yes	yes: 63% (24/38)
Hygiene at entry		yes, but regular biosecurity breaches	no	no	yes: 13% (6/47)
Rodent control		yes	no	yes	yes: 72% (34/47)
**Animal movement**					
Breeding does		purchase and sale	purchase (including does from farm A)	n.a.	89% (31/35) purchase; no sale
Fattening rabbits		purchase and sale	sale	purchase (from up to seven different breeders, including farm A)	42% (16/38)purchase; 57% (20/35) sale
Other animals on the farm		none	laying hens (*Salmonella*: negative)	broilers (*Salmonella*: negative)	n.a.
**Postmortem examinations**					
Rabbits submitted for postmortem examination	Total number of rabbits	64	1	2	n.a.
	Diagnoses (number of cases)	dysentery (15) pneumonia (12) pasteurellosis (6) otitis media (6) septicaemia (6) salmonellosis (5) intestinal coccidiosis (5) mucoid enteropathy and enteritis (3) rhinitis (3) abscesses (2) cystitis (1)	pneumonia (1) dysentery (1)	intestinal coccidiosis (1) dysentery (1)	n.a.
	Isolated pathogens (number of cases)	*Bordetella bronchiseptica* (5) *Clostridium perfringens* (4) *Escherichia coli* (19) *Klebsiella pneumoniae* (4) *Pasteurella multocida* (11) *Staphylococcus aureus* (3) *Salmonella* Typhimurium (5)	*Bordetella bronchiseptica* (1) *Clostridium perfringens* (1) *Escherichia coli* (1) *Pasteurella multocida* (1)	*Escherichia coli* (1)	n.a.

^1^ Time periods for the recording of mortality in fattening rabbits: farm A: whilst carrying out postmortem examinations; farms B and C: whilst purchasing rabbits originating from farm A, that is, the time of probable introduction of *Salmonella*.

n.a. = not applicable.

### Postmortem examination

In total, 67 meat rabbits from farms testing positive for *Salmonella* in the faecal sample were examined postmortem at the NRGK. In addition, 193 rabbits of all ages and purposes (fattening, hobby and fancy) from 181 additional husbandries, were analysed during the period of the study to determine the cause of death and also tested for *Salmonella –* thus serving as a reference group. Macroscopically altered organs were cultured aerobically/anaerobically on Columbia agar with 7% sheep blood and aerobically on bromothymol blue‐lactose agar (Oxoid; Thermo Fisher Scientific) all for 24 h at 37°C. Bacterial identification was performed using the Biotyper Matrix‐assisted Laser Desorption/Ionization‐Time of Flight‐Mass spectrometry (MALDI‐TOF‐MS) System (Bruker Daltonics). Intestinal samples were screened for the presence of *Salmonella* as described below.

### *Salmonella* detection by culture

Qualitative detection in animal faeces and organs was carried out according to ISO 6579‐1:2017.^18^ Following identification with MALDI‐TOF‐MS, *Salmonella* serotyping was carried out by the Swiss Reference Laboratory (ZOBA, University of Berne) according to the White‐Kauffmann‐Le Minor scheme.^20^

### Whole genome sequencing

Whole genome sequencing on *Salmonella* Typhimurium isolates from environmental samples from farm A (101, 122, 143, 170, 215, 257, 261, 288, 514, 540, 547, 658) and from faecal samples of farms B (N19‐2115/19‐S3101) and C (N‐19‐2269/19‐S3402) was performed using a MiSeq Illumina system (Illumina, San Diego, CA, USA)^21^, ^22^, ^23^. Briefly, the strains were grown on sheep blood agar at 37°C overnight prior to DNA isolation using the DNA blood and tissue kit (Qiagen, Hombrechtikon, Switzerland). The DNA libraries were prepared using a Nextera DNA Flex Sample Preparation Kit (Illumina). The Illumina‐reads files passed the standard quality checks using the software package FastQC 0.11.7 (Babraham Bioinformatics, Cambridge, UK) and were assembled using the Spades 3.14.1 based software Shovill 1.0.4. Core genome multilocus sequence typing (MLST) (cgMLST) analyses, including a minimum spanning tree, were done in Ridom SeqSphere+ version 5.1.0.

## RESULTS

In the first screening period, faecal samples of rabbits from 47 farms were negative for *Salmonella*, while *Salmonella* Typhimurium was detected in 3/50 farms (6%), that is, farms A, B and C (Table 1). As three rabbitries ceased their business and seven farms did not send the second sample, consecutive faecal samples of 37 farms were analysed, all with negative results.

On farm A, *Salmonella* Typhimurium was detected in breeding, restocking and fattening rabbits of all ages. Furthermore, environmental samples, including soil, hay or dust from stables, were also positive (positive faecal samples of breeding does 40/545 (7.3%), bucks 0/14 (0%), positive environmental samples 20/32 (62.5%), Table 1).

**FIGURE 1:**
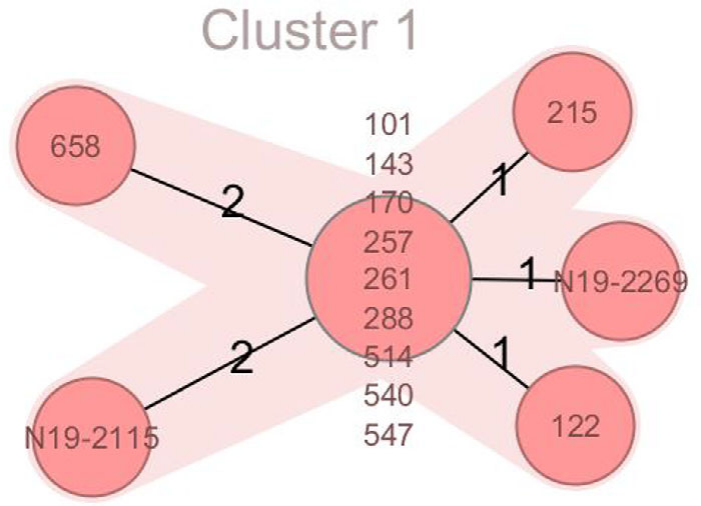
Evolutionary grouping of *Salmonella* Typhimurium isolates from rabbitry samples based on core genome multilocus sequence typing (cgMLST) allelic profiles in a minimum spanning tree Farm A: 101, 122, 143, 170, 215, 257, 261, 288, 514, 540, 547, 658; farm B: N19‐2115; farm C: N19‐2269; numbers of strains correspond to Table 1. Each circle represents an allelic profile based on sequence analysis of >1500 cgMLST target genes. The numbers on the connecting lines illustrate the number of core genes with differing alleles. The smaller the number of the differing alleles, the closer the samples are related to each other. Allele differences ≤8 are considered as genetically closely related strains which thus can be classified as evolutionary groups within phylogenetic clusters (here: one cluster)

On farms B and C, neither clinical signs compatible with salmonellosis nor increased mortality rates in the fattening units were noticed at the time of the positive sampling. Faecal samples of broilers on farm C and laying hens on farm B tested *Salmonella*‐negative. Given that both farms ceased their business, no consecutive samples were available. Farms B and C were directly linked to farm A by animal trade (Table 1). The spreading of one clone of *Salmonella* Typhimurium among the three farms was corroborated by whole genome sequencing. *Salmonella* Typhimurium isolates from the three farms showed only 1–2 allele differences and were therefore grouped within one phylogenetic cluster (Figure 1).

*Salmonella* was not detected in any of the 193 rabbits that were examined postmortem for diagnostic purposes during the study period. The motivation for the postmortem examinations was pre‐existing sickness or unsolved cause of death in individual animals or of several animals in the context of herd health monitoring. Clinical signs compatible with salmonellosis or macroscopically conspicuous tissues were observed in 114 cases. At least one of the following causes of disease per rabbit was found instead: dysentery (*n* = 66), coccidiosis (*n* = 16), dysbiosis (*n* = 8), mucoid enteropathy (*n* = 7), haemorrhagic and necrotizing typhlitis (*n* = 2), pasteurellosis (*n* = 15), pneumonia (*n* = 12), rabbit haemorrhagic disease virus type 2 (*n* = 7), encephalitozoonosis (1) and different non‐infectious diseases (*n* = 59).

## DISCUSSION

Following detection of *Salmonella* Typhimurium in commercial meat rabbits (farm A), consecutive testing revealed 94% (47/50) of Swiss commercial rabbitries to be free from *Salmonella*. The two farms additionally tested positive (B and C) were linked to outbreak farm A via animal trade (Table 1), by purchasing breeding does and/or fattening rabbits. According to cgMLST all isolates of the affected farms belonged to one cluster of genetically nearly identical *Salmonella* Typhimurium strains, displaying a single‐linkage distance of no more than two core genes (Figure 1). The combination of cgMLST and epidemiological data (both temporal and spatial link) allows the conclusion that all isolates were derived from one common ancestor. The source of infection is unknown, it is most likely that the origin was farm A, due to insufficient hygiene and rodent management, especially biosecurity breaches at the stable entries; demonstrated by various environmental samples testing positive (Table 1). On farms B and C, *Salmonella* Typhimurium was found in the pooled faecal samples of the rabbits; laying hens (farm B) and broilers (farm C) kept in separate stables tested negative. Farm A was sanitized following a programme by the Institute for Food Safety and Hygiene; farms B and C ceased farming of rabbits.

The finding of 6% of farms positive for *Salmonella* Typhimurium in this study corresponds with the clinical occurrence of *Salmonella* of less than 8% reported in other studies.^2^, ^3^, ^4^, ^7^, ^8^, ^9^ The prevalence in Switzerland might be even lower because the three positive farms correspond to a single outbreak that was detected with clinical surveillance. Among rabbits showing clinical signs compatible with salmonellosis, approximately 4% were positive for *Salmonella* in Spain with around half of them being serotyped as *Salmonella* Typhimurium^3^ – the serovar most often isolated in rabbits.^2^, ^3^, ^4^, ^6^, ^7^, ^24^ In contrast, in this and previous Swiss studies, none of the clinically conspicuous or perished rabbits tested positive for *Salmonella*.^14^, ^25^ This confirms that neither clinical signs nor postmortem findings indicative for salmonellosis are pathognomonic in rabbits but resemble different diseases of diverse origins.

The high standard of Swiss husbandry systems in terms of animal welfare is, to a certain extent, of concern regarding the transmission of infectious pathogens among rabbits housed in groups.^26^ Straw bedding is frequently used, which increases contact with faeces and urine – and thus with excreted *Salmonella*.^27^ While the present study demonstrated a low risk for Swiss rabbitries in general, this husbandry system might predispose in‐farm dispersal when coinciding with biosecurity breaches or insufficient disinfection.

Principally, strict biosecurity and regular barn disinfection help to reduce the risk of *Salmonella* contamination and spreading within a farm.^28^, ^29^ However, regular disinfection was neither carried out on *Salmonella*‐positive farms A and B, nor in 63% of the breeding units on the negative farms. Only a minority of 14% (7/50) had established a hygiene barrier (entrance and exit area with the change of clothes and handwashing facility before entering the stable). In conclusion, despite partially insufficient biosecurity measures and different risk factors for an entry of *Salmonella* into the husbandry system, the prevalence of *Salmonella* detected in this study was not higher than the one reported from other countries.

## CONFLICT OF INTEREST

The authors declare that they have no conflict of interest. The project was part of project 1.19.01 of the Swiss Federal Food Safety and Veterinary Office.

## ETHICS STATEMENT

All protocols were carried out in strict accordance with the Swiss Federal Food Safety and Veterinary Office guidelines (Animal Protection Act, Animal Protection Ordinance^15^). Approval number 30940; BE110/18.

## Data Availability

Data available on request from the authors.
